# Predictor of clinical and functional outcomes in ankle arthroscopic ligament repair with ‘all‐inside’ technique

**DOI:** 10.1002/jeo2.70464

**Published:** 2025-11-14

**Authors:** Fabrizio Forconi, Giorgio Fravolini, Chiara Muci, Maria Rosaria Matrangolo, Matteo Turchetta, Marianna Citro, Giulio Maccauro, Raffaele Vitiello

**Affiliations:** ^1^ Department of Orthopaedics and Traumatology Villa Stuart Sport Clinic FIFA Medical Center of Excellence Rome Italy; ^2^ Department of Orthopaedics and Traumatology Fondazione Policlinico Universitario Agostino Gemelli IRCCS Rome Italy; ^3^ Department of Orthopaedics and Traumatology Università Cattolica del Sacro Cuore Rome Italy

**Keywords:** all‐inside technique, arthroscopic repair, chronic ankle instability, functional outcomes, lateral ankle ligaments

## Abstract

**Purpose:**

Chronic ankle instability (CAI) is a common condition characterized by recurrent episodes of lateral instability, often due to a lesion of the ankle collateral lateral ligament complex. If untreated, CAI can lead to persistent symptoms and long‐term degenerative changes. Arthroscopic ‘all‐inside’ repair has gained traction as a minimally invasive technique offering functional and clinical benefits.

**Methods:**

This retrospective study analyzed 43 patients undergoing arthroscopic ligament repair using the all‐inside technique between 2021 and 2024. Functional outcomes were evaluated preoperatively (T0), post‐rehabilitation (T1), and at the final follow‐up (T2) using the Foot and Ankle Ability Measure (FAAM) score, VAS, and satisfaction ratings. Complications occurred were recorded during the follow up. Subgroup analyses investigated the influence of BMI, anchor use, and preoperative functional scores on outcomes.

**Results:**

Patients demonstrated significant improvement in FAAM scores (T0: 71%, T2: 95%) and pain reduction (VAS: T0: 7.67, T2: 1.00). Two‐anchor repairs yielded superior outcomes compared to single‐anchor procedures (*p* = 0.01). While higher BMI was associated with poorer outcomes (*p* = 0.04), internal bracing improved functional scores in this subgroup. Preoperative FAAM scores did not predict postoperative outcomes (*p* = 0.21).

**Conclusion:**

All‐inside arthroscopic ligament repair is a safe and effective option for CAI, providing rapid recovery, low complication rates, and excellent patient satisfaction.

**Level of Evidence:**

Level IV.

**Abbreviations**
ADL: activities of daily living
AITFL: anterior inferior tibiofibular ligament
AOFAS: American Orthopaedic Foot and Ankle score
ATFL: anterior talo‐fibular ligament
CAI: chronic ankle instability
CFL: calcaneo‐fibular ligament
EMG: electromyography
FAAM: Foot and Ankle Ability Measure
MCID: minimum clinically important difference
NRS: numerical rating scale
SEBT: Star excursion balance test
VAS: Visual Analogue Scale

## INTRODUCTION

Chronic ankle instability (CAI) is a common condition characterized by recurrent episodes of lateral ankle instability leading to repeated sprains [6]. The ankle is particularly vulnerable to injuries during sports activities and sudden movements, accounting for up to 16%–40% of all sports‐related injuries [22]. Failure to fully recover after the initial sprain may result in persistent instability, pain, and chronic symptoms that impair daily activities and quality of life [25]. The primary causes include damage to the lateral ligaments, especially the anterior talo‐fibular ligament (ATFL) and calcaneo‐fibular ligament (CFL), along with deficits in muscle strength and neuromuscular control. If left untreated, CAI may progress to early‐onset osteoarthritis [9].

Ankle sprains represent approximately 15%–30% of all musculoskeletal injuries [11], particularly affecting athletes and physically active individuals [2, 13].

In 85% of cases, sprains result from an inversion injury acting on a plantar‐flexed foot [26]. The ATFL is most commonly involved, either isolated (65%) or in combination with the CFL (20%) [8, 12, 14, 44].

Accurate diagnosis requires thorough clinical evaluation, comparing the injured and contralateral sides. Assessment includes swelling, tenderness, and range of motion, with pain usually localized near the ATFL and CFL [41].

Clinical tests, including the tilt test, anterior drawer test, and eversion test, help evaluate ligamentous damage [10, 24].

Imaging, such as X‐rays and MRI, is useful for detecting ligament injuries and associated lesions [16, 34, 47]. Additionally, functional and neurological tests, including side hop, 6 M hop test, EMG, foot lift and SEBT, may improve diagnostic accuracy. Arthroscopy is a valuable tool when imaging is inconclusive but symptoms persist [42].

Initial management of CAI is conservative; however, up to 20% of patients require surgical intervention [17].

Surgical repair of lateral ligaments has evolved since Broström's anatomical technique introduced in 1966 [3]. Modifications, such as the Broström–Gould procedure with extensor retinaculum reinforcement, report high success rates [16, 29].

The current gold standard is the modified Broström–Gould technique, with ligament reinsertion to the fibula using anchors [4, 23].

More recently, arthroscopic techniques, including percutaneous‐assisted repair [18], all‐inside ligament repair [1], and ligament reconstruction, have emerged as less invasive alternatives with encouraging outcomes [36, 39].

Vega et al. introduced the ‘all‐inside’ arthroscopic repair in 2013, reporting promising results [38].

Current evidence suggests that arthroscopic techniques provide functional and clinical outcomes comparable or superior to open surgery [19, 20].

Furthermore, ankle arthroscopy allows for simultaneous diagnosis and treatment of associated conditions, such as impingement, loose bodies, and osteochondral lesions. The purpose of our study is to assess the mid‐term clinical and functional outcomes of arthroscopic ligament repair using the all‐inside technique, as well as to identify potential predictive factors.

## MATERIAL AND METHOD

### Search strategy

A retrospective study was conducted on patients undergoing arthroscopic ankle ligament repair surgery with the all‐inside technique from March 2021 to September 2024. An informed consent was submitted to all patients participating in the study.

### Inclusion and exclusion criteria

The inclusion criteria are:

- patients with a history of CAI with persistence of symptoms 3–6 months after the traumatic event and undergoing conservative physiotherapeutic treatment.
- positive anterior drawer test.
- preoperative MRI examination confirming ligamentous injury.
- patients with a follow‐up of at least 4 months.

The following were excluded

- patients with osteochondral lesions >15 mm or noncontained lesions: these are generally considered unsuitable for arthroscopic bone marrow stimulation and instead require more invasive replacement procedures, such as osteochondral autograft transplantation (OATS) or autologous chondrocyte implantation (ACI) [15, 37, 46]. This criterion was applied to maintain cohort homogeneity and avoid confounding outcomes related to different surgical strategies.
- patients with clinical‐functional impairment that could interfere with the rehabilitation process
- patients who did not adhere to the postoperative physiotherapy protocol.

### Data collection

#### Preoperative management

All patients prior to the surgical indication have undergone a standardized physiotherapy protocol, consisting mainly of ankle muscle strengthening, stretching of the posterior chains, proprioceptive exercises and functional recovery. Subsequently, a clinical examination is repeated, evaluating residual instability with clinical tests, such as the anterior drawer test and tilt test. An ankle MRI is also repeated to assess the ligamentous status immediately preoperatively.

#### Surgical technique

All surgeries were performed by the same surgeon. The patient was placed in a supine position with the operative leg supported on a standard orthopaedic leg holder, and both the hip and knee flexed to approximately 45° to allow optimal access to the ankle joint.

A 2.7‐mm 30° arthroscope was introduced into the joint through three arthroscopic portals (anteromedial, anterolateral and accessory lateral). The joint was thoroughly inspected to assess the degree of ligamentous injury and the presence of any associated lesions (e.g., impingement, osteochondral lesions, free bodies or synovitis). The peroneal footprint was debrided, and the ligament was grasped using a 70° micro‐suture lasso or an automatic suture passer. The ligament was then reinserted using one or more knotless anchors (3.5‐mm or 4.75‐mm SwiveLock; Arthrex) positioned just distal to the peroneal insertion of the anterior inferior tibiofibular ligament (AITFL). In selected cases, synthetic augmentation with an internal brace was performed using FiberTape and SwiveLock anchors (Arthrex), following a fully arthroscopic technique.

**Figure 1:**
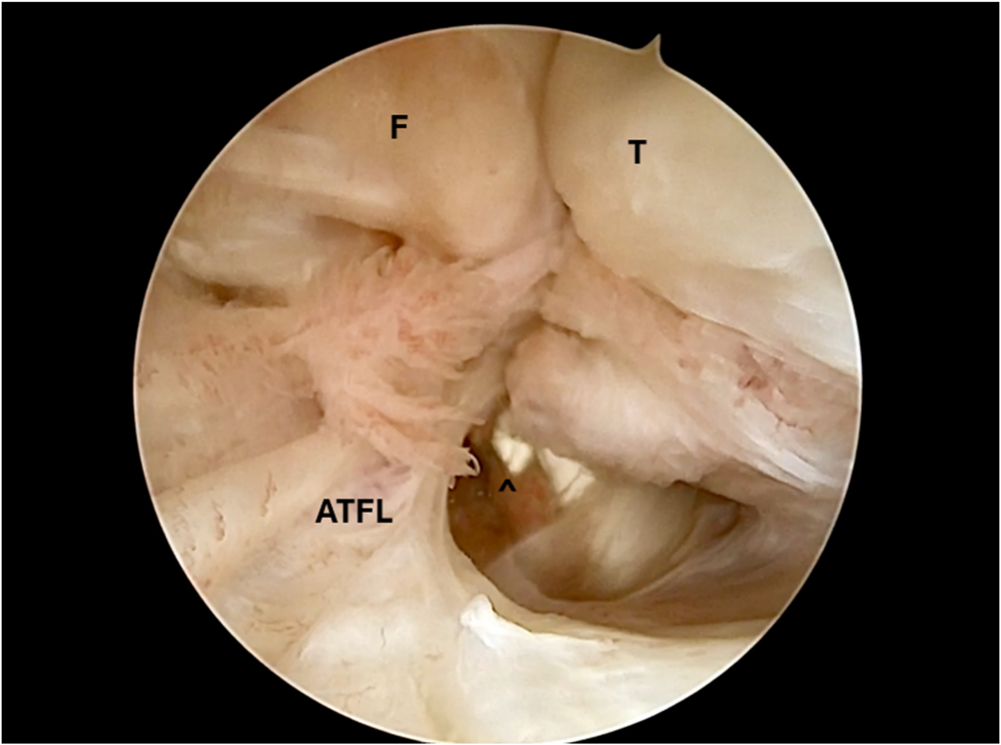
Arthroscopic view of the anterior talofibular ligament (ATFL) tear with visualization of the peroneal tendons. F, fibula; T, talus; ^, peroneal tendons.

**Figure 2:**
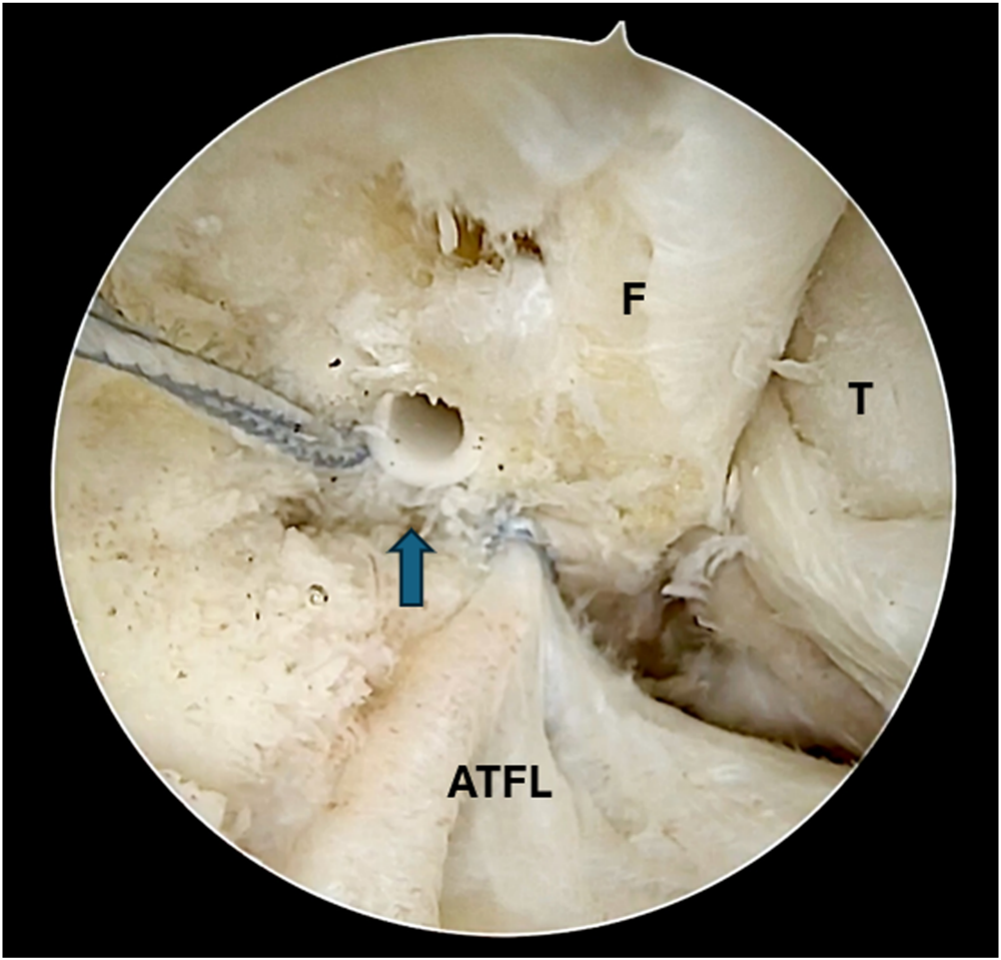
Arthroscopic repair of the ATFL using a knotless suture anchor. ATFL, anterior talofibular ligament; F, fibula; T, talus; 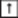, Suture anchor.

**Figure 3:**
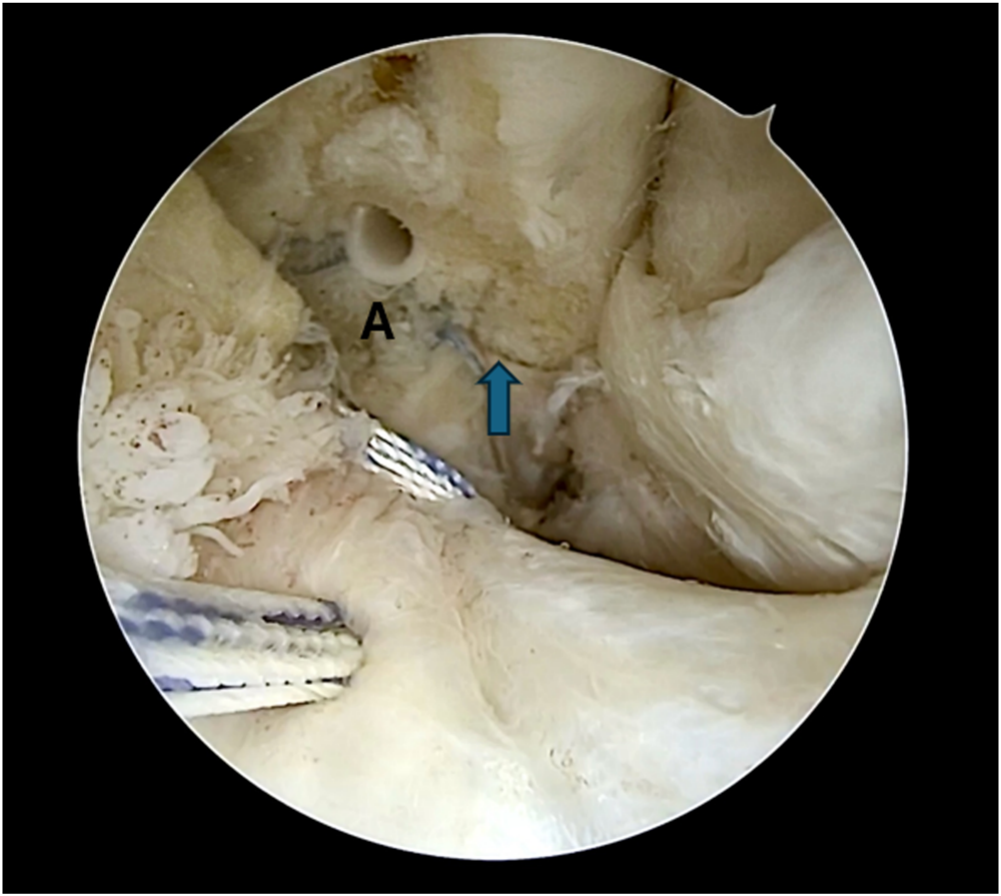
Suture tape augmentation placed in the superior bundle of the ATFL. The initial suture is visible (A), along with the drill hole for the second anchor (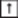). ATFL, anterior talofibular ligament.

**Figure 4:**
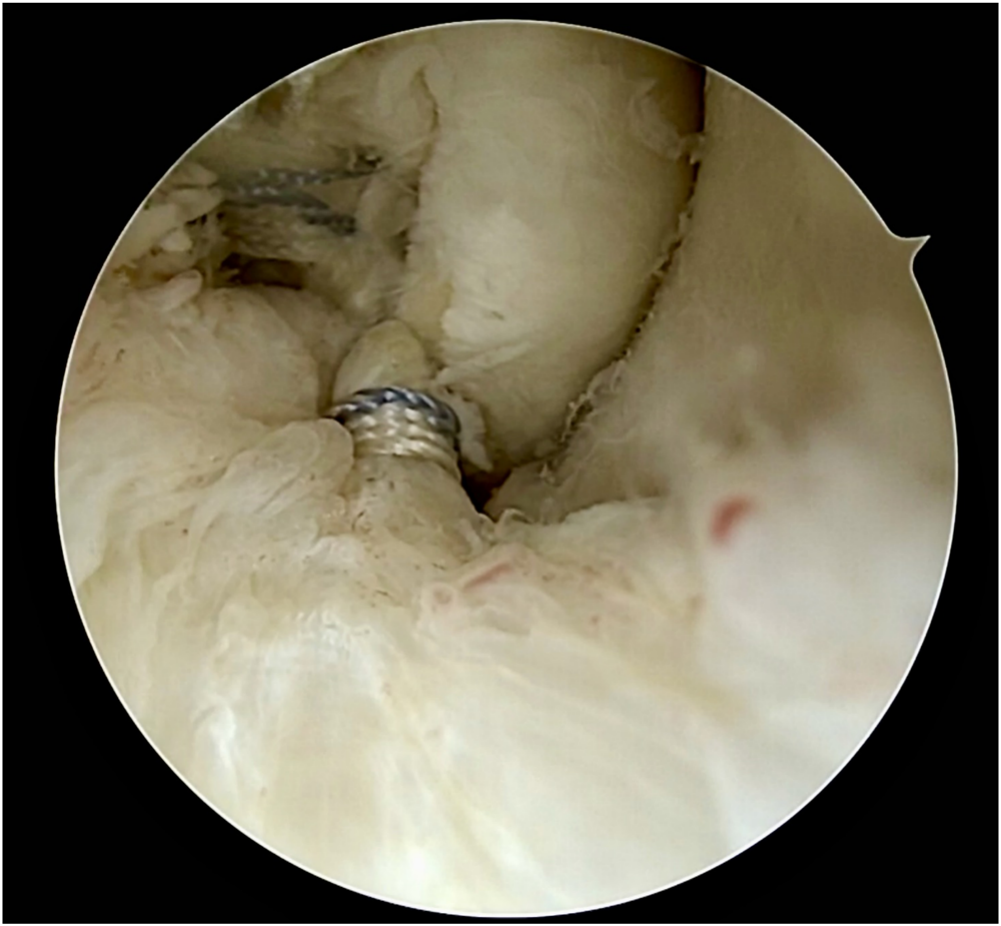
Final repair with reinforcement of both ATFL bundles and suture passage through the accessory portal for internal brace placement. ATFL, anterior talofibular ligament.

**Figure 5:**
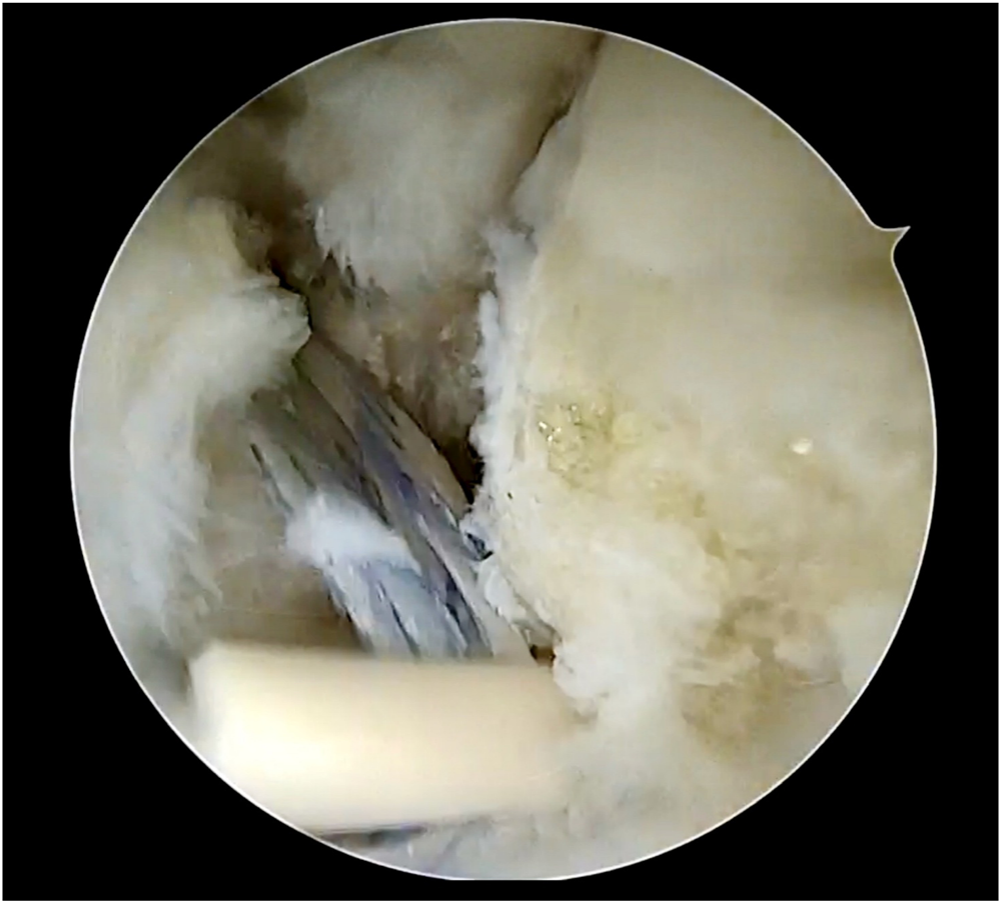
Completed internal brace construct stabilizing the anterior talofibular ligament.

Figures 1, 2, 3, 4, 5 illustrate the main steps of the arthroscopic all‐inside ligament repair technique, including anatomical landmarks and suture placement.

#### Postoperative management and follow‐up

For the first 3 weeks postoperatively, patients were allowed partial weightbearing with a Walker‐type walking boot brace. Active ankle dorsiflexion and plantarflexion exercises were encouraged every 4 hours to prevent stiffness. After 3 weeks, patients transitioned to a double‐shell stabilizing ankle brace with foam cushions and began a standard rehabilitation protocol focused on progressive muscle strengthening, proprioception training, and gradual return to full weightbearing.

The protocol concluded approximately 3 months after the surgery with the re‐athletization phase and return to sport.

A clinical evaluation with an anterior drawer test was performed at each follow‐up.

#### Evaluation scales

Patients completed questionnaires at three time points: before surgery (T0), at the end of physiotherapy, about 3 months after surgery (T1), and at the final follow‐up (T2). The Visual Analogue Scale (VAS) was employed to assess pain levels, ranging from 0 (*no pain*) to 10 (*worst pain imaginable*). A numerical rating scale (NRS) was also used to evaluate overall satisfaction with the surgery, where 0 indicated no satisfaction and 10 represented complete satisfaction.

The Foot and Ankle Ability Measure (FAAM) questionnaire was administered to evaluate functional outcomes. This self‐reported tool enables patients to rate the difficulty they experience in performing daily and sports‐related activities involving foot and ankle function. The FAAM consists of two main subscales:

Activities of Daily Living (ADL): Assesses the ability to perform routine activities such as walking, stair climbing, or standing.
Sports: Evaluates the ability to perform high‐intensity activities, such as running or jumping, in sports contexts.

Each item is scored on a scale from 0 to 4, where 0 indicates complete inability to perform the activity, and 4 indicates no difficulty [32, 33].

### Statistical analysis

Data will be described using averages and standard deviations (SD) for quantitative variables, and numbers and percentages for qualitative variables. Only two decimal places were reported, rounded up. Student's *t*‐test was used for the statistical analysis of continuous variables. A value of *p* < 0.05 is considered statistically significant.

## RESULTS

**Figure 6:**
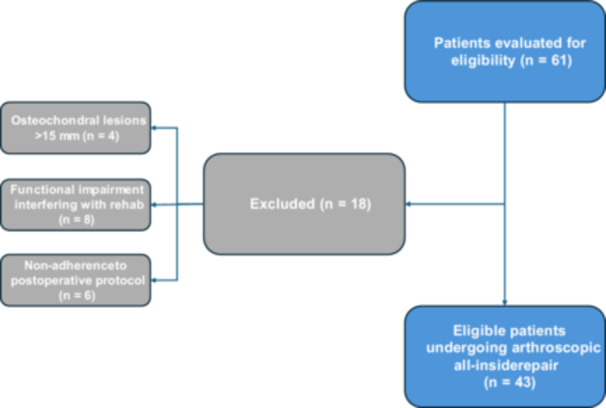
Flowchart of the patient selection process, including inclusion and exclusion criteria leading to the final study cohort.

A total of 61 patients with CAI were initially evaluated for eligibility. As detailed in Figure 6, eighteen patients were excluded due to: osteochondral lesions larger than 15 mm (*n* = 4), clinical‐functional impairment interfering with postoperative rehabilitation (*n* = 8), or nonadherence to the prescribed rehabilitation protocol (*n* = 6). Therefore, 43 patients were included in the study and underwent arthroscopic all‐inside ligament repair, comprising 19 women and 24 men. The mean age at the time of surgery was 36.2 years (SD 16.8), with an average BMI of 23.81 (SD 3.17).

All patients had a documented history of chronic lateral ankle instability. The mean number of prior ankle sprains was 4.3 (median: 3; range: 1–15), and the average time elapsed from the first reported ankle sprain to surgical intervention was 84.4 months (median: 36 months; range: 0–444 months), highlighting the chronic and recurrent nature of the condition in this cohort. These demographic and anthropometric data are summarized in Table 1.

**Table 1:** Anthropometric data of the surveyed population.

Variable	*N*
Sex	
Women	19
Men	24
Age (years)	37.72
Height (m)	1.73
Weight (kg)	70.63
BMI (kg/m^2^)	23.81
Complication	1
Previous ankle sprain	4.3
Time from the first ankle sprain to surgery (months)	84.4

Abbreviation: BMI, body mass index.

Among the study population, 31 patients maintained an active lifestyle, including 8 who participated in competitive sports. Additionally, 17 patients engaged in high‐impact sports, such as activities involving jumping, physical contact, or rapid directional changes. None of the patients had comorbidities, and the mean follow‐up period was 14.95 months (SD 8.69).

During the ligament repair procedures, two anchors were used to reinsert the anterior talofibular ligament and the calcaneofibular ligament in 23 cases. In 20 patients, internal brace reinforcement was added intraoperatively when the residual ligament tissue appeared insufficient or the mechanical stability of the repair was deemed suboptimal upon probing and dynamic testing. In 4 cases, a simultaneous deltoid ligament repair was also performed. These intraoperative findings are summarized in Table 2.

**Table 2:** Intraoperative data.

Variable	Data
Side	
Right	26
Left	17
Lateral ligament repair (number of anchors used)	
1	20
2	23
Internal brace	23
Deltoid repair	4
Associated chondral lesions	5
Complication	1

Only one patient experienced a postoperative complication, presenting with transient stupor of the superficial peroneal nerve.

Regarding pain levels, the mean VAS score decreased significantly from 7.67 (SD 1.01) preoperatively (T0) to 3.00 (SD 2.74) at the end of physiotherapy (T1) and further to 1.00 (SD 0.99) at the final follow‐up (T2). Patient satisfaction at the last follow‐up was high, with a mean score of 9.21 (SD 1.13).

Functional outcomes showed a substantial improvement in FAAM scores. The preoperative FAAM score averaged 71% (SD 0.18), increasing to 89% (SD 0.10) at midterm follow‐up and reaching 95% (SD 0.08) at the final follow‐up.

These results are summarized in Table 3.

**Table 3:** Functional outcomes.

	T0	T1	T2	T0–T1 (*p*‐value)	T1–T2 (*p* value)
VAS	7.67 ± 1.01	3 ± 2.74	1.00 ± 1.99	0.0001	0.001
FAAM ADL	0.71 ± 0.18	0.89 ± 0.10	0.95 ± 0.008	0.0001	0.0001
FAAM SPORT	0.34 ± 0.24	0.67 ± 0.19	0.89 ± 0.14	0.0001	0.0001

Abbreviations: ADL, Activities of Daily Living; FAAM, Foot and Ankle Ability Measure; VAS, Visual Analog Scale.

To investigate potential predictors of outcomes, the study population was divided into two groups based on FAAM scores at T1: Group 1 including patients with above‐average FAAM scores at T1; Group 2 including patients with below‐average FAAM scores at T1.

A paired comparison was performed between T0 versus T1 and T1 versus T2 for the FAAM‐ADL, FAAM‐SPORT, and VAS parameters. Statistically significant results were obtained for all three parameters (VAS T0–T1: 7.67 ± 1.01 vs. 3 ± 2.74; *p* = 0.0001; VAS T1–T2: 3 ± 2.74 vs. 1.00 ± 1.99; *p* = 0.001; FAAM ADL T0–T1: 0.71 ± 0.18 vs. 0.89 ± 0.10; *p* = 0.0001; FAAM ADL T1–T2: 0.89 ± 0.10 vs. 0.95 ± 0.008; *p* = 0.0001; FAAM SPORT T0–T1: 0.34 ± 0.24 vs. 0.67 ± 0.19; *p* = 0.0001; FAAM SPORT T1–T2: 0.67 ± 0.19 vs. 0.89 ± 0.14; *p* = 0.0001). These findings indicate a significant and substantial improvement in patients following surgery and physiotherapy.

A multivariate analysis was then performed, dividing the population based on the final FAAM score to identify variables influencing functional outcomes. Factors such as BMI and the number of anchors used during surgery were considered. A statistically significant difference was observed in BMI (*p* = 0.04), with Group 2, which had worse outcomes, showing a higher mean BMI of 26.3 (±2.2) compared to 22.9 (±2.6) in Group 1 (Table 4).

**Table 4:** Comparison of some anthropometric data and functional outcomes in the two groups.

	Group 1	Group 2
Age	36.2 ± 16.8	42.2 ± 12.13
BMI	22.9 ± 2.6	26.3 ± 2.2
VAS T0	7.5 ± 1.0	8.1 ± 0.75
VAS T1	2.41 ± 2.35	4.73 ± 0.99
VAS T2	0.28 ± 0.89	3.09 ± 2.8
FAAM ADL T0	0.74 ± 0.17	0.61 ± 0.18
FAAM ADL T1	0.93 ± 0.07	0.78 ± 0.09
FAAM ADL T2	0.99 ± 0.17	0.84 ± 0.32
FAAM SPORT T0	0.34 ± 0.25	0.29 ± 0.2
FAAM SPORT T1	0.70 ± 0.2	0.56 ± 0.1
FAAM SPORT T2	0.95 ± 0.07	0.70 ± 0.12
Two anchors	21	2
Satisfaction T1	8.6 ± 1.1	8 ± 1.5
Satisfaction T2	9.5 ± 0.76	8.27 ± 1.56

Abbreviations: ADL, Activities of Daily Living; BMI, body mass index; FAAM, Foot and Ankle Ability Measure; VAS, Visual Analog Scale.

Additionally, the use of two anchors was significantly associated with better outcomes (*p* = 0.01). In Group 1, 21 out of 32 patients underwent procedures using two anchors, compared to only 2 out of 11 patients in Group 2.

We conducted a multiple regression analysis to evaluate the factors influencing the final FAAM score, considering FAAM‐ADL at T0, BMI, and the number of anchors (Table 5). Significant associations were found for BMI (*p* = 0.045) and the number of anchors (*p* = 0.017), whereas FAAM‐ADL at T0 did not reach statistical significance (*p* = 0.20). This suggests that even in cases with a very low initial FAAM score, arthroscopic intervention is effective in addressing the condition.

**Table 5:** Multiple regression analysis.

Predictor	Coefficient	Estimate	Standard error	*t*‐statistic	*p* value
Constant	B_0_	1.0348	0.0985	10.5039	0
BMI	B_1_	−0.0068	0.0033	−2.0765	0.0445
N° anchor	B_2_	0.0517	0.0209	2.4754	0.0178
FAAM ADLT0	B_3_	0.0718	0.0562	1.2786	0.2086

## DISCUSSION

In recent decades, surgical techniques for the treatment of ankle instability have evolved rapidly, driven by the need to accelerate recovery, facilitate a return to sports and minimize postoperative complications. While the Broström procedure remains the gold standard [23], arthroscopy is gaining prominence in the management of CAI. More recently, the arthroscopic phase, when combined with open surgery, has been primarily used for managing associated injuries, while arthroscopy alone has proven to be a valid technique for ligament repair, with outcomes comparable to those of open surgery [21].

The all‐inside repair of the ATF and CF ligaments is a safe and reproducible technique that provides good functional outcomes and has a low rate of postoperative complications. This approach enables not only effective ligament repair but also the diagnosis and management of associated injuries [20, 23, 40].

In our study, we assessed functional outcomes and their correlation with potential risk factors. We observed that the use of a single anchor during ligament repair was associated with less favourable functional outcomes, despite significant improvements in FAAM scores from preoperative to postoperative evaluations. Specifically, the use of two anchors was significantly associated with better outcomes (*p* = 0.01), with 21 of 32 patients in Group 1 undergoing two‐anchor procedures compared to only 2 of 11 patients in Group 2. These findings are consistent with previous research, which reported higher Karlsson scores (88 ± 12 vs. 80 ± 14; *p* = 0.04), improved Tegner activity scale (5 ± 1 vs. 4 ± 1; *p* < 0.001), and greater sport participation rates (68% vs. 30%; *p* = 0.01) in patients treated with two anchors [30]. Our results highlight the consistent advantage of enhanced mechanical stabilization provided by two anchors, further emphasizing their role in achieving superior functional recovery and facilitating return to sport.

Among the 43 patients, 31 (72%) resumed an active lifestyle after surgery. Of these, 8 returned to competitive sports (e.g., swimming, volleyball, soccer, artistic gymnastics), and 17 resumed recreational high‐impact sports (e.g., running, basketball, equestrianism, climbing). The mean time to return to sport was 5.7 months (range: 4–10 months). The average self‐reported performance level, based on a 0–10 scale (where 10 indicates full return to preinjury level), was 8.9 ± 1.4. These data are detailed in Table 6.

**Table 6:** Summary of return‐to‐sport data according to sport type and participation level.

Sport type	Competitive (*n*)	Recreational (*n*)	Mean return time (months)
Soccer	2	4	5.2
Volleyball	1	2	6.0
Athletics (track)	1	2	6.7
Basketball	0	2	5.0
Running	0	4	5.5
Climbing	0	2	5.5
Horseback riding	1	1	5.5
Dance	1	0	7.0
Swimming	1	1	5.0
Cycling	0	1	6.0
Tennis	0	1	10.0
Skiing	0	1	4.0
Total	8	23	5.7

Our findings support previous evidence indicating that arthroscopic procedures enable excellent return‐to‐sport outcomes in patients with CAI. In line with prior studies reporting return to sport rates of up to 95%, with a mean return time of 12 weeks [31], our cohort showed favourable recovery even among competitive and high‐impact sport participants. Factors such as younger age, high preinjury activity levels, and sport‐related injuries are associated with more favourable outcomes, while increased BMI, advanced age, and postoperative immobilization with a lower‐leg cast negatively influence return to sport success [5, 45]. These findings emphasize the efficacy of arthroscopic techniques in achieving a timely and successful return to sport, even in individuals involved in high‐impact or competitive activities, and highlight the importance of individualized rehabilitation and early mobilization protocols in optimizing sport‐specific functional recovery.

Our study revealed that preoperative FAAM scores (T0) did not significantly influence postoperative functional outcomes, as no difference in T0 values was observed between the two groups (*p* = 0.21). This finding suggests that even patients presenting with poor baseline functional scores can achieve excellent results with the all‐inside arthroscopic technique. The absence of significant predictive value for T0 aligns with the growing evidence that other factors, such as BMI, surgical technique, and postoperative management, play more critical roles in determining outcomes. The literature addressing preoperative functional status as a predictor of outcomes in CAI is remarkably sparse. While previous studies have identified factors such as age, sex, type of injury, and postoperative immobilization as significant predictors of functional recovery, none have explicitly explored the impact of preoperative functional scores [5]. This lack of evidence underscores the novelty of our findings and highlights the need for further research to validate the observation that poor baseline function does not preclude successful surgical outcomes.

Another notable finding in our study was the impact of BMI on postoperative outcomes. Patients with higher BMI demonstrated poorer functional results, as reflected in lower FAAM scores, a trend that aligns with limited but emerging evidence in the literature. While BMI has often been considered a potential risk factor for suboptimal outcomes, studies have shown that the all‐inside arthroscopic Broström procedure for chronic lateral ankle instability remains effective even in patients with a BMI ≥ 30 kg/m². Functional outcomes, including AOFAS, VAS, and Foot Function Index scores, have been reported to show no significant differences between patients with elevated BMI and those with BMI < 30 kg/m² [7]. These findings suggest that this minimally invasive technique provides reliable stabilization across a range of BMI values, though challenges in rehabilitation for higher BMI patients should still be considered.

Our findings suggest a trend indicating that patients with a BMI > 24 who underwent internal bracing achieved higher postoperative FAAM scores (94% vs. 89%). However, this difference did not reach statistical significance. While elevated BMI is a recognized risk factor for ankle sprains and instability (e.g., higher BMI observed in patients with lateral ankle sprains and neuromuscular deficits associated with instability), its role in CAI prognosis remains unclear [28, 43]. Moreover, BMI has not been shown to significantly correlate with CAI outcomes [48]. This trend highlights the potential value of internal bracing in optimizing functional recovery in patients with elevated BMI, warranting further investigation.

Despite these variations in functional outcomes, patient satisfaction levels remained high across the cohort, even among individuals with less favourable objective measures (Group 1: T1 = 8.6; T2 = 9.5, Group 2: T1 = 8; T2 = 8.27). This aligns with the concept that improvements perceived by patients may not always correspond directly to measured functional scores. Indeed, the minimum clinically important difference (MCID) for FAAM subscales has been shown to vary considerably among patients, emphasizing the subjective nature of outcome evaluation [27, 35].

Our study demonstrated that FAAM‐ADL at T0 did not exhibit a significant correlation with FAAM‐ADL at T2 (*p* = 0.20), in contrast to BMI (*p* = 0.045) and the number of anchors used (*p* = 0.017), which were found to be significant predictors of functional outcomes. This suggests that preoperative functional limitations do not necessarily impair the final recovery of joint function or the overall clinical improvement following surgery. These findings provide important insights into the potential for successful rehabilitation regardless of the patient's initial functional status. Notably, this aspect has not been extensively investigated in the existing literature, underscoring the need for further research to confirm these observations and to explore additional factors that may contribute to postoperative recovery and long‐term functional outcomes.

This study has several strengths. First, all surgical procedures were performed by a single experienced surgeon, ensuring consistency in technique. Second, a standardized postoperative rehabilitation protocol was followed for all patients, reducing variability in recovery trajectories. Additionally, the sample size and follow‐up period were sufficient to provide meaningful insights into medium‐term outcomes. Although the mean follow‐up duration in our cohort provides valuable insights into medium‐term outcomes, long‐term follow‐up is necessary to evaluate the durability of the surgical repair. Specifically, future studies should aim to assess the recurrence of instability and the development of degenerative changes such as post‐traumatic osteoarthritis, which may emerge beyond the current follow‐up period.

However, there are some limitations. The small number of patients with elevated BMI restricts the ability to generalize findings related to this subgroup. Our findings suggest a trend indicating improved outcomes in patients with BMI > 24 who received internal bracing; however, the small sample size of this subgroup limits statistical power and prevents definitive conclusions. These observations should be interpreted as preliminary and hypothesis‐generating, warranting validation in larger, adequately powered cohorts. Furthermore, the retrospective nature of the study introduces inherent limitations, including the possibility of selection bias and the lack of randomization. These factors may influence the internal validity of the results. Additionally, the absence of a control or comparison group—such as patients treated with the traditional open Broström‐Gould technique—precludes definitive conclusions regarding the superiority or equivalence of the all‐inside arthroscopic approach. Future prospective randomized controlled trials are needed to address these comparative questions. Finally, the retrospective design of the study introduces the possibility of selection bias, which could influence the results.

## CONCLUSION

All‐inside arthroscopic repair with knotless anchors is an effective, minimally invasive option for treating CAI, enabling rapid recovery and return to sport. The use of two anchors significantly improves functional outcomes compared to one anchor, emphasizing the importance of enhanced mechanical stabilization. BMI emerged as a potential risk factor for poorer outcomes, but internal bracing showed promising results in improving functional recovery in this subgroup. Importantly, poor preoperative functional scores did not affect postoperative outcomes, highlighting the versatility of this technique even in patients with severe baseline impairment.

## AUTHOR CONTRIBUTIONS

All of the authors as well as the responsible authorities have approved the contents of this paper and have agreed to the KSSTA policies. Each named author has substantially contributed to conducting the underlying research and draughting this manuscript.

## ACKNOWLEGEMENTS

Open access funding provided by UCSC Università Cattolica del Sacro Cuore.

## CONFLICT OF INTEREST STATEMENT

The authors declare no conflicts of interest.

## ETHICS STATEMENT

Ethical review and approval were waived for this study due to the retrospective nature of the research and the use of de‐identified patient data, in accordance with the policies of the Institutional Review Board. Informed consent was obtained from the patient included in the study.

## Data Availability

The data that support the findings of this study are available from the corresponding author upon reasonable request.
